# A Case of Postoperative Recurrent Lumbar Disc Herniation Conservatively Treated with Novel Intradiscal Condoliase Injection

**DOI:** 10.1155/2022/3656753

**Published:** 2022-02-15

**Authors:** Toru Funayama, Yusuke Setojima, Yosuke Shibao, Hiroshi Noguchi, Kousei Miura, Fumihiko Eto, Kosuke Sato, Mamoru Kono, Tomoyuki Asada, Hiroshi Takahashi, Masaki Tatsumura, Masao Koda, Masashi Yamazaki

**Affiliations:** ^1^Department of Orthopaedic Surgery, Faculty of Medicine, University of Tsukuba, 1-1-1 Tennoudai, Tsukuba, 3058575 Ibaraki, Japan; ^2^Department of Orthopaedic Surgery, Kenpoku Medical Center Takahagi Kyodo Hospital, 1006-9, Ageho-cho, Kamitetsuna, Takahagi, 318-0004 Ibaraki, Japan; ^3^Department of Orthopaedic Surgery and Sports Medicine, Tsukuba University Hospital Mito Clinical Education and Training Center/Mito Kyodo General Hospital, 3-2-7 Miyamachi, Mito, 310-0015 Ibaraki, Japan

## Abstract

Although postoperative recurrent lumbar disc herniation (rec-LDH) is uncommon, it is a challenging situation that requires revision surgery when conservative treatment fails. Recently, an agent inducing chemical dissolution of the nucleus pulposus using condoliase has been approved as a novel intradiscal treatment for LDH. To date, no evidence has been reported regarding its effectiveness in the treatment of postoperative rec-LDH. A 25-year-old man with a history of LDH in L4/5, who underwent transforaminal full endoscopic lumbar discectomy when he was 17 years old, complained of severe pain radiating to his left leg since 1 month. The straight leg-raising test was limited to 25° on the left side. Lumbar T2-weighted magnetic resonance imaging (MRI) showed intracanal, left-sided transligamentous disc herniation at L4/5 with high-signal intensity. Because the conservative treatment with oral analgesics and selective left L5 nerve root block failed, the patient requested intradiscal condoliase injection instead of revision surgery. There were no adverse events reported after the condoliase treatment, and the pain radiating to the left leg improved within 2 weeks. A lumbar MRI performed 2 months after treatment revealed that the disc herniation had significantly decreased in size. The straight leg-raising test examined 3 months after treatment was negative. In this case, the disc herniation was of the transligamentous type and showed a high-signal intensity on T2-weighted MRI which could be suitably treated by condoliase injection therapy. This case report is the first to suggest that intradiscal condoliase injection could be a useful and novel conservative treatment option to treat postoperative rec-LDH.

## 1. Introduction

Postoperative recurrent lumbar disc herniation (rec-LDH) is a challenging condition [1] and accounts for 3-9.3% of cases after transforaminal full endoscopic lumbar discectomy (FELD) [2–4], similar to that of microendoscopic discectomy [5]. Due to compression of the traversing spinal nerve root, revision surgery should be considered for rec-LDH when conservative treatment fails [1].

An agent that induces the chemical dissolution of the nucleus pulposus using condoliase (Hernicore®, Kaken Pharmaceutical Co.) [6] was recently approved as a novel intradiscal treatment for LDH [7, 8]. However, no evidence has been reported regarding its effectiveness in the treatment of postoperative rec-LDH in the English literature to date.

## 2. Case Presentation

**Figure 1:**
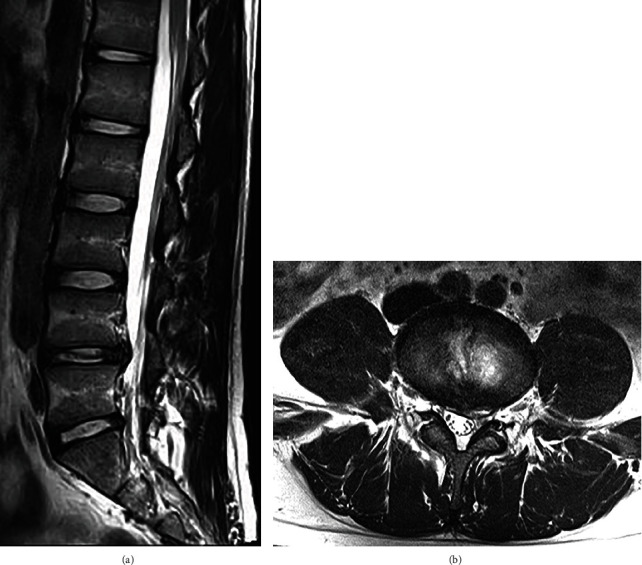
Magnetic resonance imaging before transforaminal full endoscopic lumbar discectomy 8 years ago. A (a) sagittal section and (b) horizontal section of the L4/5 showed subligamentous lumbar disc herniation.

A 25-year-old man with a history of LDH in L4/5, who had previously undergone transforaminal FELD at our institute at the age of 17 years (Figures 1(a) and 1(b)), complained of severe pain radiating to his left leg since 1 month. He had received a selective left L5 nerve root block at another hospital 10 days prior. Although his leg pain disappeared immediately after the block, it returned on the same night. He was an office worker at a sales position but had to be absent from work due to the pain. Physical examination revealed normal patellar and Achilles tendon reflexes on both sides, and manual muscle testing and lower limb sensations were normal. The pain was absent when he was seated at rest but intensified when he stood up or walked. The straight leg-raising test (SLRT) was positive and limited to 25° on the left, whereas the femoral nerve stretch test was negative on both sides. The Japanese Orthopaedic Association (JOA) score was 16 out of 29 points. The JOA Back Pain Evaluation Questionnaire (JOABPEQ) scores out of 100 for low back pain, lumbar function, walking ability, social life function, and mental health were 0, 0, 0, 8, and 41, respectively. The visual analog scale (VAS) scores out of 10 for low back pain, leg pain, and leg numbness were 9 each.

**Figure 2:**
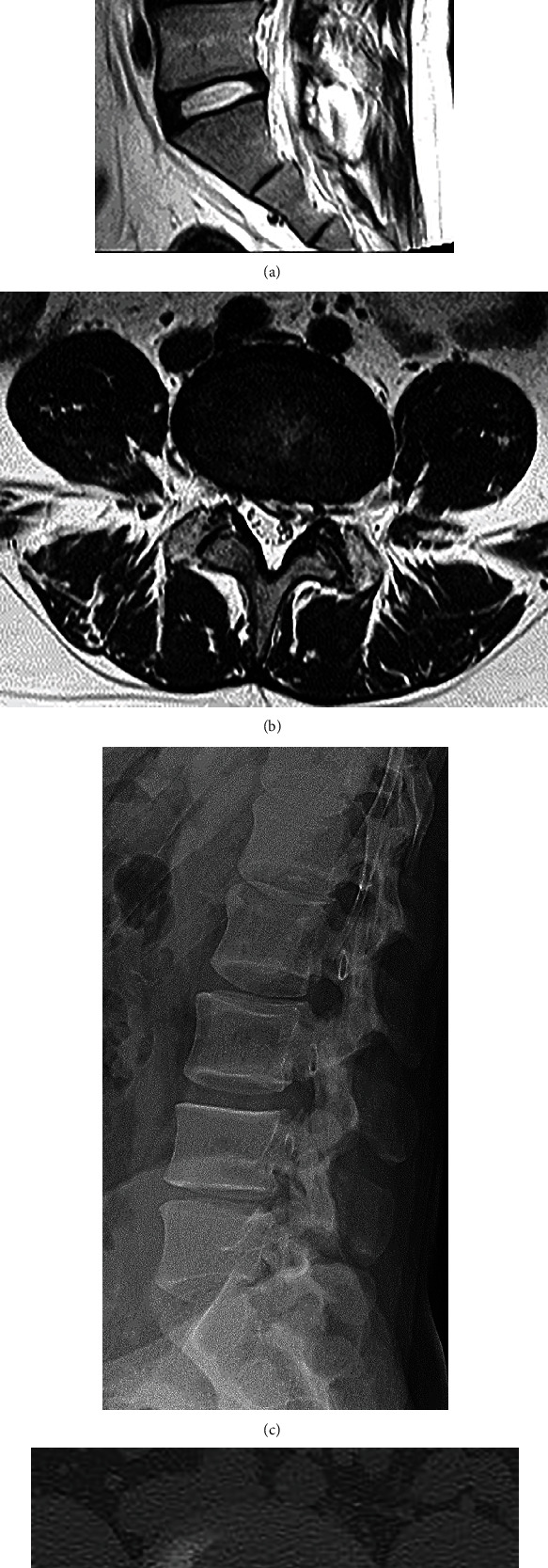
Spinal imaging before intradiscal condoliase injection. A (a) sagittal section and (b) horizontal section of the L4/5 on T2-weighted magnetic resonance imaging showed intracanal, left-sided transligamentous disc herniation with high-signal intensity. (a) The degree of affected disc degeneration was grade III according to the Pfirrmann classification [9]. (c) Standing radiography revealed that the L4/5 disc height was slightly diminished as compared to the other discs. (d) Computed tomography showed no bony stenosis at L4/5.

Lumbar T2-weighted MRI showed intracanal, left-sided transligamentous disc herniation at L4/5 with high-signal intensity (Figures 2(a) and 2(b)). The degree of affected-disc degeneration was grade III according to the Pfirrmann classification [9] (Figure 2(a)). Standing radiography revealed that the L4/5 disc height had diminished slightly as compared to the other discs (Figure 2(c)). Computed tomography showed no bony stenosis (Figure 2(d)).

**Figure 3:**
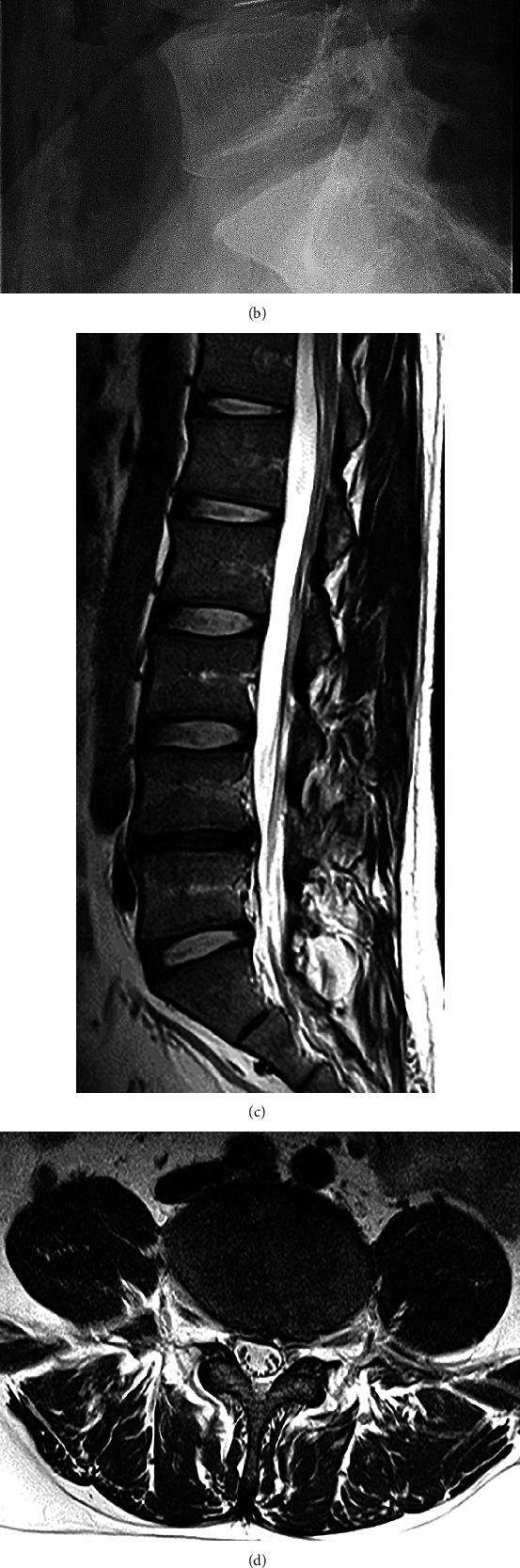
Condoliase injection procedure and findings after treatment. In the prone position, a 12 cm spinal needle was inserted from the left lateral side under fluoroscopy, advanced to the center of the L4/5 disc (a, b), and the condoliase was injected. Two months after treatment, lumbar T2-weighted magnetic resonance imaging showed that the disc herniation at L4/5 had significantly decreased in size (c, d). Although progression of the degree of affected-disc degeneration was observed (c), standing radiography revealed that the L4/5 disc height was the same as that before treatment (e).

Because oral pregabalin (300 mg/day), tramadol hydrochloride/acetaminophen (37.5 mg/325 mg; 3 tablets/day) and selective nerve root block showed poor analgesic effects in the patient, the L5 radiculopathy was considered resistant to conservative treatment. Although we informed the patient that there was no evidence regarding the effectiveness of intradiscal condoliase injection in the treatment of postoperative rec-LDH, the patient requested it instead of revision surgery. In the prone position, a 12 cm spinal needle was inserted from the left lateral side under fluoroscopy, advanced to the center of the L4/5 disc, and 1.25 U of condoliase dissolved in 1.2 mL of physiological saline was injected (Figures 3(a) and 3(b)).

No adverse events were reported after the treatment, and the pain radiating to the left leg improved within 2 weeks. Two months after treatment, the lumbar T2-weighted MRI showed that the disc herniation at L4/5 had significantly decreased in size (Figures 3(c) and 3(d)). Although progression of the degree of affected-disc degeneration was observed (Figure 3(c)), standing radiography revealed that the L4/5 disc height was the same as that before treatment (Figure 3(e)). Three months after treatment, the SLRT was negative on both sides, and the JOA score improved to 26 out of 29 points. The JOABPEQ scores out of 100 for low back pain, lumbar function, walking ability, social life function, and mental health were 100, 75, 86, 54, and 57, respectively, and the VAS scores out of 10 for low back pain, leg pain, and leg numbness were 2, 3, and 3, respectively, thus showing significant improvements in all parameters.

## 3. Discussion

Condoliase injected into an intervertebral disc specifically dissolves glycosaminoglycans, which constitute proteoglycans, the main component of the nucleus pulposus [6]. This reduces the water-holding capacity of the proteoglycans, reduces the pressure inside the intervertebral disc, lowers the pressure on the nerve root from the hernia, and leads to improvement in the lower extremity and low back pain [7].

A previous study observed significant analgesic effects in 85.4% of the patients [10], improved lumbar function, and improved quality of life as compared to the placebo treatment in intracanal LDH cases [8]. Additionally, a recent report of condoliase injection showed good results for lateral LDH [11]. Other studies concluded that condoliase injection seems to be the most effective treatment option for the transligamentous type of LDH [12] and herniation showing high-signal intensity on T2-weighted MRI [12, 13].

This is the first report on the effectiveness of condoliase injections in treating postoperative rec-LDH and avoiding the need for revision surgery. In this case, conservative therapy with oral medications and selective nerve root block was unsuccessful. However, intradiscal condoliase injection proved effective, achieving an analgesic effect soon after treatment, which was not inferior to revision surgery [14]. The disc herniation of the present case was of the transligamentous type and showed high-signal intensity on the T2-weighted MRI, which was suitable for condoliase injection therapy for nonoperated LDH [12, 13].

Although more findings from comparative studies or large case series including various surgical methods other than transforaminal FELD are needed, this report suggests that intradiscal condoliase injection could be a useful and novel conservative treatment option with a possibility of avoiding the need for revision surgery in postoperative rec-LDH.
